# In vitro investigations on extracellular proteins secreted by *Aphanomyces invadans*, the causative agent of epizootic ulcerative syndrome

**DOI:** 10.1186/s13028-017-0347-3

**Published:** 2017-11-09

**Authors:** Muhammad Majeed, Gokhlesh Kumar, Sarah Schlosser, Mansour El-Matbouli, Mona Saleh

**Affiliations:** 1 Clinical Division of Fish Medicine, University of Veterinary Medicine, Veterinärplatz 1, 1210 Vienna, Austria; 2 VetCore Facility for Research, University of Veterinary Medicine, Vienna, Austria

**Keywords:** *Aphanomyces invadans*, Epizootic ulcerative syndrome, LC–MS/MS, Oomycetes, Proteases, Virulence

## Abstract

**Background:**

Proteases produced by many microorganisms, including oomycetes, are crucial for their growth and development. They may also play a critical role in disease manifestation. Epizootic ulcerative syndrome is one of the most destructive fish diseases known. It is caused by the oomycete *Aphanomyces invadans* and leads to mass mortalities of cultured and wild fish in many countries. The areas of concern are Australia, China, Japan, South and Southeast Asian countries and the USA. Extracellular proteases produced by this oomycete are believed to trigger EUS pathogenesis in fish. To address this activity, we collected the extracellular products (ECP) of *A. invadans* and identified the secreted proteins using SDS-PAGE and mass spectrometery. *A. invadans* was cultivated in liquid Glucose-Peptone-Yeats media. The culture media was ultra-filtered through 10 kDa filters and analysed using SDS-PAGE. Three prominent protein bands from the SDS gel were excised and identified by mass spectrometery. Furthermore, we assessed their proteolytic effect on casein and immunoglobulin M (IgM) of rainbow trout (*Oncorhynchus mykiss*) and giant gourami (*Osphronemus goramy*). Antiprotease activity of the fish serum was also investigated.

**Results:**

BLASTp analysis revealed that the prominent secreted proteins were proteases, mainly of the serine and cysteine types. Proteins containing fascin-like domain and bromodomain were also identified. We could demonstrate that the secreted proteases showed proteolytic activity against the casein and the IgM of both fish species. The anti-protease activity experiment showed that the percent inhibition of the common carp serum was 94.2% while that of rainbow trout and giant gourami serum was 7.7 and 12.9%, respectively.

**Conclusions:**

The identified proteases, especially serine proteases, could be the potential virulence factors in *A. invadans* and, hence, are candidates for further functional and host–pathogen interaction studies. The role of identified structural proteins in *A. invadans* also needs to be investigated further.

## Background

Infection with *Aphanomyces invadans*, also known as epizootic ulcerative syndrome (EUS), is an OIE-listed disease [1]. It has historically caused mass mortalities in cultured and wild fish in Asia and Oceania and recently in North America and Africa. An EUS-like condition was first reported from Japan in farmed Ayu (*Plecoglossus altivelis*) [2, 3] and later from Australia. Initial symptoms are described as focal haemorrhages on fish skin with variable size of ulcerative lesions that may progress to necrotizing dermatitis resulting in deep dermal ulcer [4, 5]. The economic impact of this disease is huge. It has caused an estimated loss of US $110 million only in few countries of Asia–Pacific region during late 80’s and early 90’s [6]. Clinical EUS has not been reported from Europe so far, but *A. invadans* has been detected in imported ornamental fish species from different non-European countries [7].

EUS is a water-borne disease caused by *A. invadans*. It is a slow growing, thermolabile [8] and non-septate microorganism [9]. It lacks reproductive structures and belongs to the class Oomycota (water moulds) and order Saprolegniales [6, 10]. Oomycetes, including *Aphanomyces* and *Saprolegnia* species, have caused some of the most devastating fish diseases [11]. About 125 fish species have been confirmed to be affected by *A. invadans* [12] and the number is gradually increasing [13]. Recently, the mitochondrial genomes of *A. invadans* and *Aphanomyces astaci* were assembled and annotated, which provides good basis for further functional studies and development of diagnostic methods [14].

Proteases are vital for fungal physiology and development and digesting external proteins required for fungal growth [15]. However, many infectious microorganisms, including fungi and oomycetes, produce extracellular proteases or effector proteins that initiate the disease process [15].

Involvement of microbial proteases in disease development in humans and using these proteases as targets for development of therapeutic agents against such diseases has also been reported [16]. An important strategy in drug development is to focus on natural inhibitors of disease-involved proteases to counter their effects. Although natural antiproteases are present in every organism, they can be repressed by the pathogens, hence triggering disease development.

Extracellular proteases produced by the oomycete *A. invadans* have been suggested to play a part in EUS pathogenesis. Proteases have also been reported to affect the immune response of the host and support the invasion of the hyphae in host musculature [17]. In this study, we aimed at identifying *A. invadans*’ secreted proteases using SDS-PAGE and mass spectrometery and investigating their proteolytic activity against casein and fish immunoglobulin M (IgM). The results of this study suggest that the secreted proteases may be considered as potential virulence factors of *A. invadans* in EUS.

## Methods

### Oomycete culture and zoospore production

**Fig. 1:**
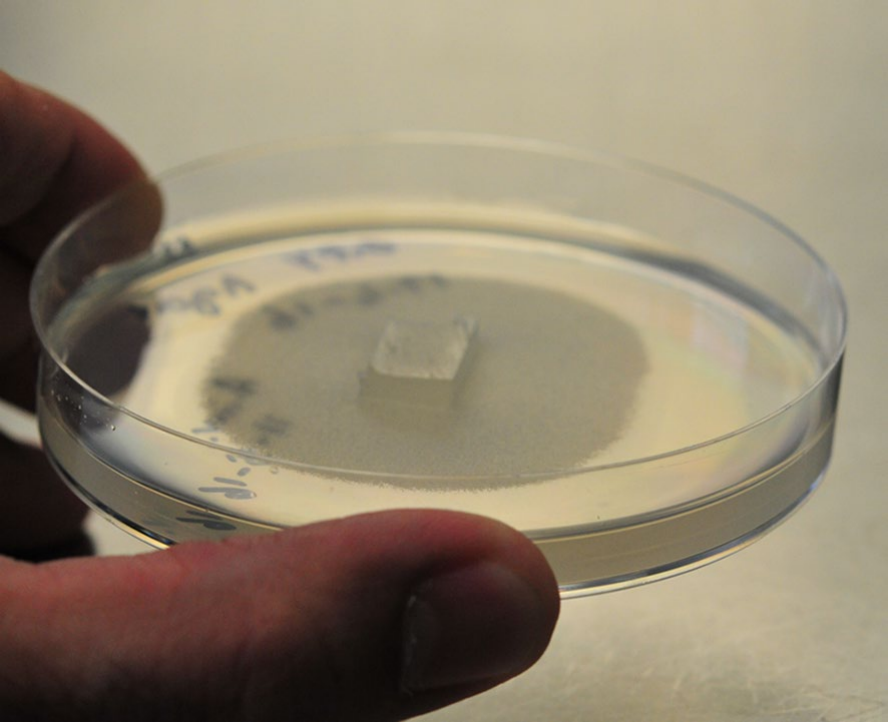
*Aphanomyces invadans* culture. The PG-1 agar plate shows the growth of *Aphanomyces invadans*

*Aphanomyces invadans* NJM 9701 was cultured on peptone glucose-1 (PG-1) agar (Fig. 1). Fungal plugs (6–7 cm^2^) were cut and placed on new PG-1 plates every 6–7 days as subculture and maintained at 25 °C. In parallel, the fungal plugs were grown at 25 °C for 6–7 days in glucose-peptone-yeast (GPY) broth. These fungal plugs were collected and placed in filtered autoclaved pond water (FAPW) pH 7.2 for 12–24 h at 22 °C for zoospore production [6]. Zoospores were observed under light microscope.

### Germination of zoospores and isolation of extracellular products

FAPW suspension containing zoospores was transferred to double strength GPY broth in 1:1 ratio and placed at 25 °C for 6–7 days for the germination of zoospores [6]. The contents of the plates were then pooled into a 50 mL tube and centrifuged to remove the fungal hyphae. The soluble fraction as a source of extracellular products was collected and subsequently filtered using 10 kDa Amicon Ultra-15 filters (Sigma, Germany) at 3220 g for 15 min. Retentate was collected as final extracellular product (hereafter referred as ECP).

### SDS-PAGE

ECP samples (10 µL) in triplicate were subjected to 12% SDS-PAGE as described by Laemmli [18] and Kumar et al. [19] with some modifications. Samples were mixed with 2 × SDS gel-loading buffer and heated at 100 °C for 5 min. Electrophoresis was carried out at 60 V for 4 h (BIO-RAD). The gel was stained with Coomassie blue G-250. Three bands of interest were excised manually from the gel. After washing and destaining with methanol: acetic acid mixture, the proteins were reduced with dithiothreitol and alkylated with iodoacetamide [20]. In-gel digestion was performed with trypsin (Promega) for 8 h at 37 °C [21]. Peptides were extracted, dried and redissolved in 0.1% aqueous TFA prior to LC–MS injection.

### nanoLC–MS/MS analysis

Sample pre-concentration and desalting was accomplished with a 5 mm Acclaim PepMap μ‑Precolumn (300 µm inner diameter, 5 µm particle size, and 100 Å pore size, Dionex). For sample loading and desalting 2% ACN in ultra pure water with 0.05% TFA was used as a mobile phase with a flow rate of 5 µL/min.

Separation of peptides was performed on a 25 cm Acclaim PepMap C18 column (75 µm inner diameter, 3 µm particle size, and 100 Å pore size) with a flow rate of 300 nL/min. The gradient started with 4% B (80% ACN with 0.1% formic acid) and increased to 35% B in 120 min. It was followed by a washing step with 90% B. Mobile Phase A consisted of ultra pure water with 0.1% formic acid. For mass spectrometric analysis the LC was directly coupled to a high resolution quadrupole time of flight mass spectrometer (Triple TOF 5600 + , Sciex) via nano electrospray source.

For information dependent data acquisition (IDA runs) MS1 spectra were collected in the range of 400–1500 *m/z*. The 25 most intense precursors with charge state 2–4, which exceeded 100 counts per second, were selected for fragmentation for 250 ms. MS2 spectra were collected in the range 100–1800 *m/z* for 110 ms. Precursor ions were dynamically excluded from reselection for 12 s. The nano-HPLC system was operated by Chromeleon 6.8 (Dionex) and the MS by Analyst Software 1.6 (Sciex). Database searches of raw files were processed with ProteinPilot Software version 5.0 (Sciex). The UniProt and NCBI database (Release 08_2016) was restricted to *A. invadans.* After having the result of mass spectrometer analysis, a BLASTp analysis was done using Uniprot and NCBI database.

### Protease activity experiment using casein as substrate

To confirm the protease activity of the ECP, caseinolytic activity was assessed according to the method described by Cupp-Enyard [22]. Briefly, reaction mixture containing 0.5 mL ECP was added to 5 mL 0.65% casein solution in PBS pH 7.5 and incubated for 10 min at 37 °C. The reaction was stopped by adding 5 mL trichloroacetic acid (TCA). A blank tube was run in parallel. Appropriate volume of ECP was then added in both tubes to bring the final volume to 1 mL, incubated for 30 min at 37 °C and filtered afterwards. 2 mL filtrate was mixed with 5 mL Na_2_CO_3_ solution (500 mM) followed by the addition of 1 mL Folins-Ciocalteu’s (FC) reagent and incubated for 30 min at 37 °C. Tyrosine standard vials were also prepared side by side. The contents of these tubes were then filtered, the amount of tyrosine released was measured at 660 nm and protease activity in units/mL was calculated according to the formula by Cupp-Enyard [22].

### Protease activity experiment using IgM as substrate

Rainbow trout (*Oncorhynchus mykiss*) and giant gourami (*Osphronemus goramy*) were anaesthetized using ethyl 3-aminobenzoate methanesulfonate (Sigma, Darmstadt, Germany) (MS-222; 100 mg/L) and their blood was collected through caudal vein puncture using non-heparinized sterile syringe with 23G needle (B|Braun, Maria Enzersdorf, Austria). Serum was obtained by spinning the blood at 2000 g for 10 min. IgM precipitation was performed using ammonium sulfate as per the protocol described by Hay and Westwood [23]. Further, the activity of extracellular proteases was tested against fish IgM using dot blot analysis as per the protocol described by Jiang et al. [24] with slight modification. Briefly, 20 µL ECP as a source of secreted proteases were incubated overnight at 10 °C with 20 µL IgM (400 ng) from both fish species separately. EDTA (metalloproteinase inhibitor, 10 mM), E-64 (a cysteine protease inhibitor, 10 µM) and phenylmethanesulfonyl fluoride (PMSF) (serine protease inhibitor, 1 mM) were used to confirm that the proteases in the ECP were responsible for the observed proteolytic activity and to identify the classes of proteases. IgM with GPY broth and with water were separately incubated as negative controls. After overnight incubation, 2.5 µL sample volume from each reaction was spotted onto nitrocellulose membranes, dried for 45–50 min at room temperature and blocked with 5% dry milk for 30–40 min. Membranes were then accordingly dipped in anti-rainbow trout and anti-giant gourami monoclonal antibodies (Aquatic Diagnostics) for 10 min and washed four times with PBS for 15 min. Remaining IgM was detected using goat-anti-mouse HRP conjugated antibody (BIO-RAD).

### Antiprotease activity of fish serum

The capacity of the fish serum to degrade the proteases was tested according to the method described by Zuo and Woo [25] with slight modifications. Briefly, sera from rainbow trout, giant gourami and common carp (*Cyprinus carpio*) were collected as described above. 10 µL test serum from each fish species was incubated with 100 µL trypsin (100 µg/mL) (bovine pancrease type I, Sigma, Germany) at 25 °C along with 110 µL PBS as negative control, 10 µL PBS and 100 µL trypsin as positive control and 10 µL serum and 100 µL PBS as serum blank. After 30 min incubation 1 mL 0.25% casein was added and incubated for 15 min at 25 °C. The reaction was stopped by adding 500 µL 10% TCA. Samples were then centrifuged to remove the precipitates. The absorbance (optical density; OD) of the supernatant was measured at 280 nm and anti-protease activity was calculated [17, 25]: Reference value (RV) = Absorbance of the positive control − Absorbance of the negative control Test value (TV) = Absorbance of the test sample − Absorbance of the serum blank Percent inhibition = ((RV − TV)/RV) × 100.

## Results

### Zoospores, proteases and the ECP

**Fig. 2:**
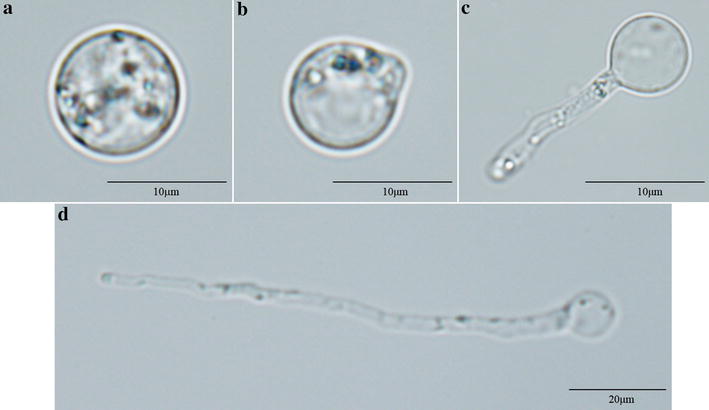
Different phases of germinating spores of *Aphanomyces invadans*. **a** Spore having thick cell wall. **b** Spore starts to germinate as the development of a small outgrowth (a bud-like structure) is seen. **c** Newly-formed small hyphae. **d** A long thread like structure (hyphae)

Thick-walled zoospores were observed under microscope (Fig. 2). Germinating zoospores produced hyphae that covered the entire surface area of the plate in 7–8 days. Filtering the soluble fraction using 10 kDa filters yielded 200–250 µL ECP.

### Identified proteins

**Fig. 3:**
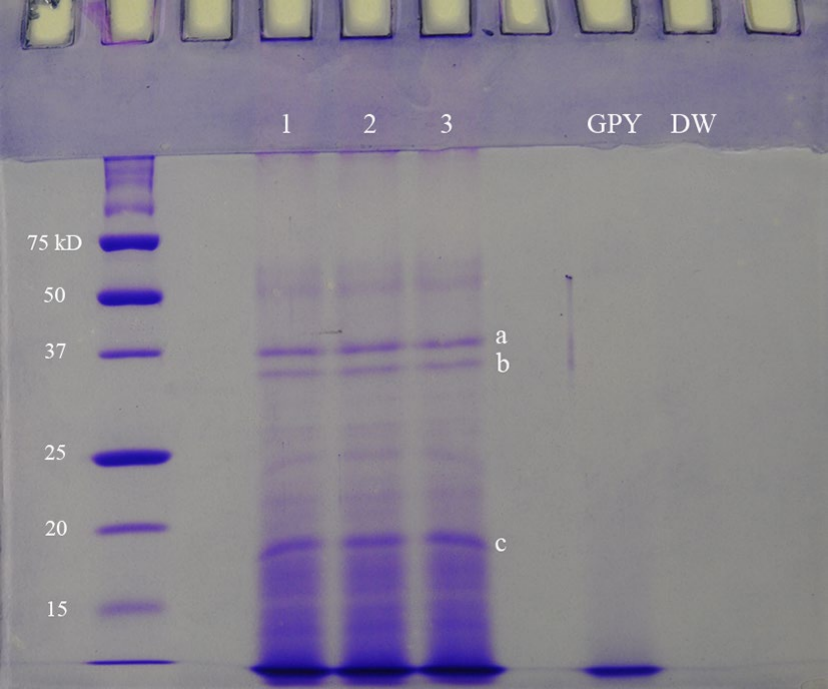
SDS-PAGE profile of extracellular products of *Aphanomyces invadans*. Lane 1, 2 and 3: Triplicate ECP samples showing three prominent protein bands (a, b and c). These protein bands were identified as proteases by mass spectrometer and subsequent BLASTp analysis. GPY broth and distilled water (DW) were used as negative controls

SDS-PAGE showed three prominently expressed protein bands (Fig. 3). Using nano LC-MS/MS and subsequent BLASTp analyses, a total of 5, 13 and 12 proteins were identified in bands a, b and c, respectively (Table 1). These proteins were identified mainly as proteases including serine and cysteine. Proteins containing fascin-like domain and bromodomain were also identified.

**Table 1:** List of hypothetical proteins identified in extracellular product of *Aphanomyces invadans*

Band	NCBI accession	Total ProtScore	Sequence coverage (%)	Confident peptides/protein	Conserved domain
a	gi|673043857	24	28.6	22	Peptidase_S8_S53 super family
	gi|673054889	20.96	21.6	15	Fascin super family
	gi|673023204	6	8.6	3	Trypsin_2
	gi|673025822	4	4.6	2	Peptidase_S8_S53 super family
	gi|673031752	2.04	7.5	6	Peptidase_S8_S53 super family
b	gi|673043857	17.69	25.8	22	Peptidase_S8_S53 super family
	gi|673054889	15.29	19.1	9	Fascin super family
	gi|673031752	14	20.6	10	Peptidase_S8_S53 super family
	gi|673040359	9.29	27.5	5	Trypsin_2
	gi|673039729	7.19	9.00	4	Peptidase_S8_S53 super family
	gi|673049945	6	8.5	4	Glyco_hydro_1 super family
	gi|673023204	6	8.6	3	Trypsin_2
	gi|673036800	4	5.3	2	Peptidase_C1A
	gi|673036298	4	8.1	3	—^a^
	gi|673036612	3	4.9	2	Peptidase_C1 super family
	gi|673033024	2	0.3	2	Bromodomain super family
	gi|673022270	2	2.5	2	S1-P1_nuclease
	gi|673021356	2	2.5	3	Peptidase_C1
c	gi|673054889	28	39.8	35	Fascin super family
	gi|673038963	14.04	15.3	8	Beta-lactamase
	gi|673043857	14	15.2	11	Peptidase_S8_S53 super family
	gi|673039719	8	13.5	4	Peptidase_S8_S53 super family
	gi|673050561	6	5.3	3	Peptidase_C1
	gi|673040359	4.03	12.3	2	Trypsin_2
	gi|673050209	4	13.1	2	—^a^
	gi|673028596	4	13.8	2	—^a^
	gi|673025822	4	4.6	2	Peptidase_S8_S53 super family
	gi|673039729	6	4.2	3	Peptidase_S8_S53 super family
	gi|673051517	2	2.8	2	Peptidase_C1A
	gi|673033024	2	0.3	2	Bromodomain super family

Conserved domain of each protein was identified using NCBI database. No conserved domain was found against the proteins/proteases marked with^a^

### Protease activity experiments

**Fig. 4:**
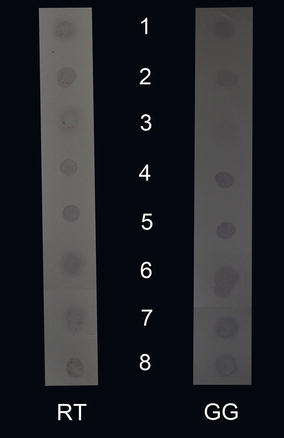
Protease activity of *Aphanomyces invadans* secreted proteases against IgM of rainbow trout (RT) and giant gourami (GG). Spot 1: IgM was degraded by proteases as no dark spot is seen. Spot 2: IgM was intact when proteases were heat inactivated and upon addition of anti-antibodies and the substrate produced a dark spot. 3: ECP and IgM both were heat inactivated hence produced no dark spot. Spots 4 and 5: IgM was intact in GPY media and distilled water respectively hence produced dark spots. Spot 6: EDTA, Spot 7: E-64 protease inhibitor and Spot 8: phenylmethanesulfonyl fluoride inhibited the proteases hence dark spots are seen

Activity of protease sample using casein as substrate was calculated which was found to be 0.206 units/mL. Dot blot results showed that the proteases were able to degrade the IgM of both fish species (Fig. 4 spot-1) however inactivated proteases were unable to proteolyze the IgM (Fig. 4 spot-2). No color was seen when both IgM and proteases were heat inactivated (Fig. 4 spot-3). IgM in GPY and distilled water were used as positive controls (Fig. 4 spots-4 and 5, respectively). The protease inhibitors EDTA, E-64 and PMSF inhibited the protease activity (Fig. 4 spots-6, 7 and 8, respectively).

### Total anti-protease activity

Total anti-protease activity assay showed that the percent inhibition of the carp serum was 94.2% while that of rainbow trout and giant gourami serum was 7.7 and 12.9%, respectively.

## Discussion

*Aphanomyces* were initially known as parasites of plants, small invertebrates and crustaceans. Probably the first case reported of fish being infected by *Aphanomyces* species was from guppy (*Poecilia reticulata*), Maxican blind cave (*Astyanax mexicanus*), a hybrid of platy (*Platypoecilus maculatus*) and swordtail (*Xiphophorus hellerii*) [26]. *Aphanomyces*, along with other oomycetes, e.g. *Saprolegnia* species have caused severe losses to aquaculture industry till to-date. From an evolutionary point of view it seems that the pathogenesis of plant oomycetes is mediated by effectors, while host invasion by animal pathogenic oomycetes is facilitated by expansion of proteases [24]. Many infectious microorganisms require proteases for replication or use proteases as virulence factors to cross proteinaceous barrier within the host. Proteases secreted by the fish oomycete *Saprolegnia parasitica* are suggested to be involved in attacking the host tissue and paving the way for further penetration of the fungus into the fish [24]. In the present study, we investigated *A. invadans*’ secreted proteases using SDS-PAGE and mass spectrometery followed by BLASTp analysis. 30 peptides belonging to eight major protein domains have been identified (Table 1). Identified proteases containing Trypsin_2 domain (gi|673023204 and gi|673040359) show sequence similarity to *Aphanomyces astaci*, *Saprolegnia parasitica*, *Saprolegnia diclina*, *Achlya hypogyna* and *Thraustotheca clavata*. Dubovenko et al. [27] have reported that most of the fungal species that have trypsin or similar-to-trypsin peptidases genes are pathogenic to animals, plant and fungi and suggested them as possible markers of pathogenicity. Extracellularly secreted trypsin-like proteases of *A. invadans* might have a similar role.

Identified protein with fascin-like domain (gi|673054889) was found in all three bands. Members of fascin-like domain are actin bundling proteins required for motility and invasion in *Rickettsia conorii* and *Listeria* [28, 29]. Actin polymerization is the main driving force for cell locomotion. It is used by the bacteria e.g. *Listeria* sp. and *Shigella* sp., and viruses e.g. vaccinia for intracellular and intercellular movements [30, 31]. We suggest that this protein may also be required for actin-bundling in *A. invadans* and has a conserved role in adhesion, mobility and invasion in oomycetes as in other pathogens [28–31]. However, the functional mechanisms of protein members of the fascin-like domain and their role in adhesion, motility and invasion remain largely unknown in oomycetes.

Bromodomains are chromatin-associated proteins and are important mediators of gene expression. Such proteins are reported to be crucial in viability and virulence of protozoan parasites [32] and are reported to be essential in the pathogenesis of *Plasmodium falciparum* [33]. S1–P1 nucleases have been reported to be involved in the degradation of the genetic material in programmed cell death in plants [34]. In our study, the identified proteases that belong to bromodomain (gi|673033024) and S1–P1_nuclease domain (gi|673022270) might also have pathogenic as well as structural role in *A. invadans* as in other pathogens [32, 33]. Beta-lactamase, mostly produced by bacteria, have an indirect role in infection. These are mostly used against the broad-spectrum beta-lactam antibiotics and provide resistance [35]. Beta-lactamase (gi|673038963) identified in the ECP of *A. invadans* suggests that these proteins might have a similar role against beta-lactam broad-spectrum antibiotics and may promote resistance.

Glycoside hydrolase (GH) enzymes are one of the largest families of enzymes produced by the fungi to break down plant cell wall [36]. One GH enzyme, XEG1, from plant pathogenic oomycete *Phytophthora sojae* has been reported as a major virulence factor in soybean [37]. As animals, including fish, do not contain large amounts of cellulose on their skin, extracellular secretion of GH domain enzyme (gi|673049945) from an animal pathogen, *A. invadans*, mainly reflects the adaptation to nutrient supply [38].

The C1 group of peptidases belongs to the papain-like cysteine proteases. This type of proteases is important for parasites for their virulence and tissue and cell invasion [39]. Many plant pathogenic bacteria produce cysteine effectors to proteolyze the host substrate to alter the plant physiology [40]. Fish parasitic oomycete, *S. parasitica*, has been reported to secrete cysteine proteases, among others, that could function in virulence [41]. Cystein proteases identified in our study (gi|673036800, gi|673036612, gi|673021356, gi|673050561 and gi|673051517) may also be important in *A. invadans* virulence.

However, the most prominent identified protein in our results is the serine type peptidase as 10 out of 30 identified peptides belong to Peptidase_S8 domain with active serine motif. The S8 family has a catalytic triad similar to that found in trypsin-like proteases. BLASTp analysis against NCBI database suggested that the identified hypothetical proteins gi|673043857, gi|673025822, gi|673031752, gi|673039729 and gi|673039719 have significant sequence similarity to peptidase_S8 domain from a range of animal and plant parasitic oomycetes including *A. astaci*, *S. parasitica*, *S. diclina*, *P. sojae*, *P. parasitica*, *Plasmopara halstedii* and *A. hypogyna* and nonpathogenic saprophytic oomycete *Teinoptila clavata*. One of the important classes of this protease domain is subtilisin-like serine protease. These proteases have long been implicated as virulence factor in bacteria and true fungi [11]. A serine protease, SPRG_14567, secreted by oomycete *S. parasitica* has been reported to be able to degrade the rainbow trout IgM [24]. Our results suggest that at least one of these identified serine type proteases is also involved in degradation of rainbow trout and giant gourami’s IgM.

Involvement of extracellular serine protease of a nematophagous fungus *Lecanicillium psalliotae* in degradation of casein has been reported [42]. We found that the extracellular proteases secreted by *A. invadans* have actively degraded the casein. Consequently, we were encouraged to also investigate the proteolytic activity of these proteases against fish IgM. Fish depends mainly on its innate immunity as fundamental defense against antigens [43, 44]. IgM is the first antibody to be produced after an antigen attacks the host. These are also the natural antibodies of the innate immune system presented as an initial defense component. These immunoglobulins immediately recognize the invading pathogens and aim to destruct them [45, 46]. Banfield and Kamoun [47] have reported that degradation of IgM of rainbow trout by the proteases of *S. parasitica* is critical in order to cause the disease saprolegniasis. Jiang et al. [24] have also reported that at least one of the proteases produced by *S. parasitica* has the ability to degrade the IgM of rainbow trout, which suggests that the oomycete has the ability to manipulate and conquer the initial immune response of the fish.

We tested the secreted proteases of *A. invadans* against IgM of rainbow trout (*Oncorhynchus mykiss*) and giant gourami (*Osphronemus goramy*) and found that the proteases were able to degrade the IgM of both fish species. These are involved in competing against the initial immune defense of the fish thus enables the oomycete to invade the host and play a part in EUS pathogenesis. Lilley et al. [48] suggested that the virulence of pathogenic *A. invadans* may be attributed to production of proteolytic enzymes.

Dot blot results showed that EDTA, PMSF and E-64 inhibited the protease activity of ECP suggesting that metalloproteinase, cysteine and serine proteases were secreted by *A. invadans*. These findings are in agreement with the results obtained by mass spectrometery and BLASTp analysis.

Fish plasma contains enzymes, or antiproteases, that play important role in degrading the proteases of invading pathogens. These are mainly α-1 antiprotease, α-2 antiplasmin and α-2 macroglobulin [25, 49]. These antiproteases have the ability to degrade the extracellular proteases secreted by pathogens hence restricting the entry of the pathogen. Results of our antiprotease activity experiment showed that common carp had the highest protease inhibition capacity as compared to rainbow trout and giant gourami. These findings could explain the resistance of common carp to infection with *A. invadans* [50, 51] and the susceptibility of the other two species tested. Consequently, the virulence of *A. invadan*s could be reduced if protease secretion is affected or strong anti-proteases are used.

Our findings offer a baseline for further investigations on the role of the secreted proteases in EUS pathogenesis using genome editing tools, such as CRISPR/Cas9 system, and pave the way for the development of novel drugs to control the disease.

## Conclusions

The ECP of *A. invadans* was collected and secreted proteins were identified using SDS-PAGE and mass spectrometery. BLASTp analysis revealed that the prominent secreted proteins were proteases, mainly the serine and cysteine type. These secreted proteases degraded the casein and the initial defense component (IgM) of rainbow trout and giant gourami. Dot blot results showed that inhibition of these proteases using various protease inhibitors resulted in reduced proteolytic activity. Our findings suggest that these extracellular proteases could potentially be involved in EUS virulence mechanisms. Hence, they may experimentally be targeted and explored for development of novel controlling methods and therapeutics for EUS.
